# Association between institutional case volume and mortality following thoracic aorta replacement: a nationwide Korean cohort study

**DOI:** 10.1186/s13019-020-01204-0

**Published:** 2020-06-29

**Authors:** Karam Nam, Eun Jin Jang, Jun Woo Jo, Jae Woong Choi, Minkyoo Lee, Ho Geol Ryu

**Affiliations:** 1 Department of Anesthesiology and Pain Medicine, Seoul National University Hospital, Seoul National University College of Medicine, 101 Daehak-ro, Jongno-gu, Seoul, 03080 Republic of Korea; 2 Department of Information Statistics, Andong National University, Andong, Gyeongsangbuk-do Korea; 3 Department of Statistics, Kyungpook National University, Daegu, Korea; 4 Department of Thoracic and Cardiovascular Surgery, Seoul National University Hospital, Seoul National University College of Medicine, Seoul, Korea

**Keywords:** Case volume, Surgical prognosis, Thoracic aorta replacement, Volume-outcome relationship

## Abstract

**Background:**

The inverse relationship between case volume and postoperative mortality following high-risk surgical procedures have been reported. Thoracic aorta surgery is associated with one of the highest postoperative mortality. The relationship between institutional case volume and postoperative mortality in patients undergoing thoracic aorta replacement surgery was evaluated.

**Methods:**

All thoracic aorta replacement surgeries performed in Korea between 2009 and 2016 in adult patients were analyzed using an administrative database. Hospitals were divided into low (< 30 cases/year), medium (30–60 cases/year), or high (> 60 cases/year) volume centers depending on the annual average number of thoracic aorta replacement surgeries performed. The impact of case volume on in-hospital mortality was assessed using the logistic regression.

**Results:**

Across 83 hospitals, 4867 cases of thoracic aorta replacement were performed. In-hospital mortality was 8.6% (191/2222), 10.7% (77/717), and 21.9% (422/1928) in high, medium, and low volume centers, respectively. The adjusted risk of in-hospital mortality was significantly higher in medium (odds ratio [OR], 1.56; 95% confidence interval [CI], 1.16–2.11, *P* = 0.004) and low volume centers (OR, 3.12; 95% CI, 2.54–3.85, *P* < 0.001) compared to high volume centers.

**Conclusions:**

Patients who had underwent thoracic aorta replacement surgery in lower volume centers had increased risk of in-hospital mortality after surgery compared to those in higher volume centers. Our results may provide the basis for minimum case volume requirement or regionalization in thoracic aorta replacement surgery for optimal patient outcome.

## Background

The association between case volume and patient outcome in complex surgical procedures have been reported consistently [1–3]. The mechanism behind the relationship is unclear but proposed explanations include accumulated experience at the individual or institutional level and preferential referral, which in turn may lead to increased case volume and improved outcomes. Numerous reports regarding the inverse relationship between case volume and postoperative mortality after high-risk procedures have led to debates/discussions concerning regionalization or efficient allocation of medical resources [4–8].

Thoracic aorta replacement surgery is one of the most complex surgical procedures requiring meticulous perioperative care with a reported 30-day mortality rate ranging from 5 to 10% [9, 10]. Moreover, serious postoperative complications including paraplegia or stroke are not uncommon, and thus, there is still room for improvement in patient outcomes despite recent improvement [10, 11]. Institutions with higher volume or more experience are likely to have a system or protocol regarding high-risk surgical procedures and the management thereafter [1]. However, the impact of institutional case volume in thoracic aorta replacement surgery on patient outcome has not been evaluated.

The aim of the study was to determine the relationship between institutional case volume and postoperative mortality in patients undergoing thoracic aorta replacement surgery. A population-based, retrospective observational study was performed by analyzing the National Health Insurance Service (NHIS) database in Korea to evaluate the case volume effect in thoracic aorta replacement surgery.

## Methods

The study design was a nationwide population-based retrospective observational study. The study protocol was determined exempt from review by the Institutional Review Board of Seoul National University Hospital due to the retrospective study design and the de-identified nature of the database.

### Study population and data collection

Data from the NHIS database which covers more than 97% of Koreans was used for analysis [12, 13]. All adult cases of isolated thoracic aorta replacement surgery performed between January 2009 and December 2016 in Korea were analyzed using the procedure codes for ascending aorta, aortic arch, and descending thoracic aorta replacement surgeries. Preoperative comorbidities were identified using the International Classification of Diseases, 10th revision (ICD-10) codes. Codes for emergent surgery and perioperative red blood cell transfusion were also extracted from the NHIS database. In-hospital, 1-year, and cumulative all-cause mortality were also collected. The institutional case volume was defined as the annual average number of thoracic aorta replacement surgeries performed during the study period. Centers were classified as low (< 30 cases/year), medium (30–60 cases/year), or high volume centers (> 60 cases/year) according to case volume of the center.

### Study endpoints and statistical analysis

The primary outcome was in-hospital mortality after thoracic aorta replacement surgery according to the institutional case volume. Secondary outcomes included 1-year mortality and cumulative all-cause mortality.

Continuous data were expressed as mean (standard deviation) or median (interquartile range) where appropriate and categorical data as number (%). To compare patient characteristics and preoperative comorbidities, the one-way analysis of variance or the Kruskal-Wallis test was used for continuous variables and the χ^2^ test for categorical variables.

Logistic regression was performed to analyze the risk of in-hospital and 1-year mortality. Multivariable logistic regression was performed to adjust for extracted relevant variables including patient characteristics, preoperative comorbidities, and the year of surgery without applying any variable selection method. The amount of perioperative red blood cell transfusion was categorized: 0–1, 2–3, 4–5, and ≥ 6 units.

Cox proportional hazards model was used to compare the risk of cumulative all-cause mortality according to institutional case volume. The log-minus-log plot was used to check whether the proportional hazards assumption was met. Kaplan-Meier survival curves were also plotted.

All analyses were performed using SAS (ver. 9.4; SAS Institute, Cary, NC) and R (ver. 3.6.1; R Development Core Team, Vienna, Austria). A *P* value under 0.05 was considered statistically significant.

## Results

Overall, 4867 cases of thoracic aorta replacement surgery were performed across 83 centers in Korea between January 2009 and December 2016. Thoracic aorta replacement surgery was performed on 1928 patients in 72 low volume centers, 717 patients in 5 medium volume centers, and 2222 in 6 high volume centers. Baseline characteristics according to case volume strata are presented in Table 1. Patients in high volume centers were older and had higher rate of comorbidities such as hypertension, hyperlipidemia, diabetes, and atrial fibrillation compared to patients in low and medium volume centers. In addition, descending thoracic aorta replacement and combined (2 or more of the 3 segments) thoracic aorta replacement were more frequently performed in high volume centers compared to lower volume centers (Table 1).

**Table 1:** Patient characteristics and preoperative comorbidities according to case volume

	Low volume (< 30 cases/year, *n* = 1928)	Medium volume (30–60 cases/year, *n* = 717)	High volume (> 60 cases/year, *n* = 2222)	*P*
Age (years)	61.9 (14.4)	60.6 (14.7)	63.2 (13.3)	< 0.001
Female	969 (50.3%)	340 (47.4%)	844 (38.0%)	< 0.001
Extracardiac arteriopathy	250 (13.0%)	77 (10.7%)	298 (13.4%)	0.174
Renal impairment	31 (1.6%)	7 (1.0%)	29 (1.3%)	0.430
Chronic lung disease	623 (32.3%)	239 (33.3%)	745 (33.5%)	0.695
Hypertension	1151 (59.7%)	397 (55.4%)	1446 (65.1%)	< 0.001
Hyperlipidemia	382 (19.8%)	154 (21.5%)	618 (27.8%)	< 0.001
Diabetes mellitus	150 (7.8%)	42 (5.9%)	193 (8.7%)	0.049
Atrial fibrillation	66 (3.4%)	26 (3.6%)	133 (6.0%)	< 0.001
Angina pectoris	351 (18.2%)	154 (21.5%)	521 (23.5%)	< 0.001
Recent MI	42 (2.2%)	7 (1.0%)	37 (1.7%)	0.101
History of PCI	15 (0.8%)	8 (1.1%)	26 (1.2%)	0.429
Congestive heart failure	157 (8.1%)	69 (9.6%)	180 (8.1%)	0.405
Emergent surgery	104 (5.4%)	27 (3.8%)	70 (3.2%)	0.001
Perioperative RBC Transfusion (units)	4 (3–5)	3 (2–4)	3 (2–5)	< 0.001
Surgery site				< 0.001
Ascending aorta	805 (41.8%)	312 (43.5%)	330 (14.9%)	
Aortic arch	185 (9.6%)	30 (4.2%)	81 (3.7%)	
Descending thoracic aorta	283 (14.7%)	144 (20.1%)	672 (30.2%)	
Combined	655 (34.0%)	231 (32.2%)	1139 (51.3%)	

Data are presented as number (%), mean (standard deviation), or median (interquartile range)

*MI* Myocardial infarction, *PCI* Percutaneous coronary intervention, *RBC* Red blood cell

### In-hospital mortality

**Fig. 1:**
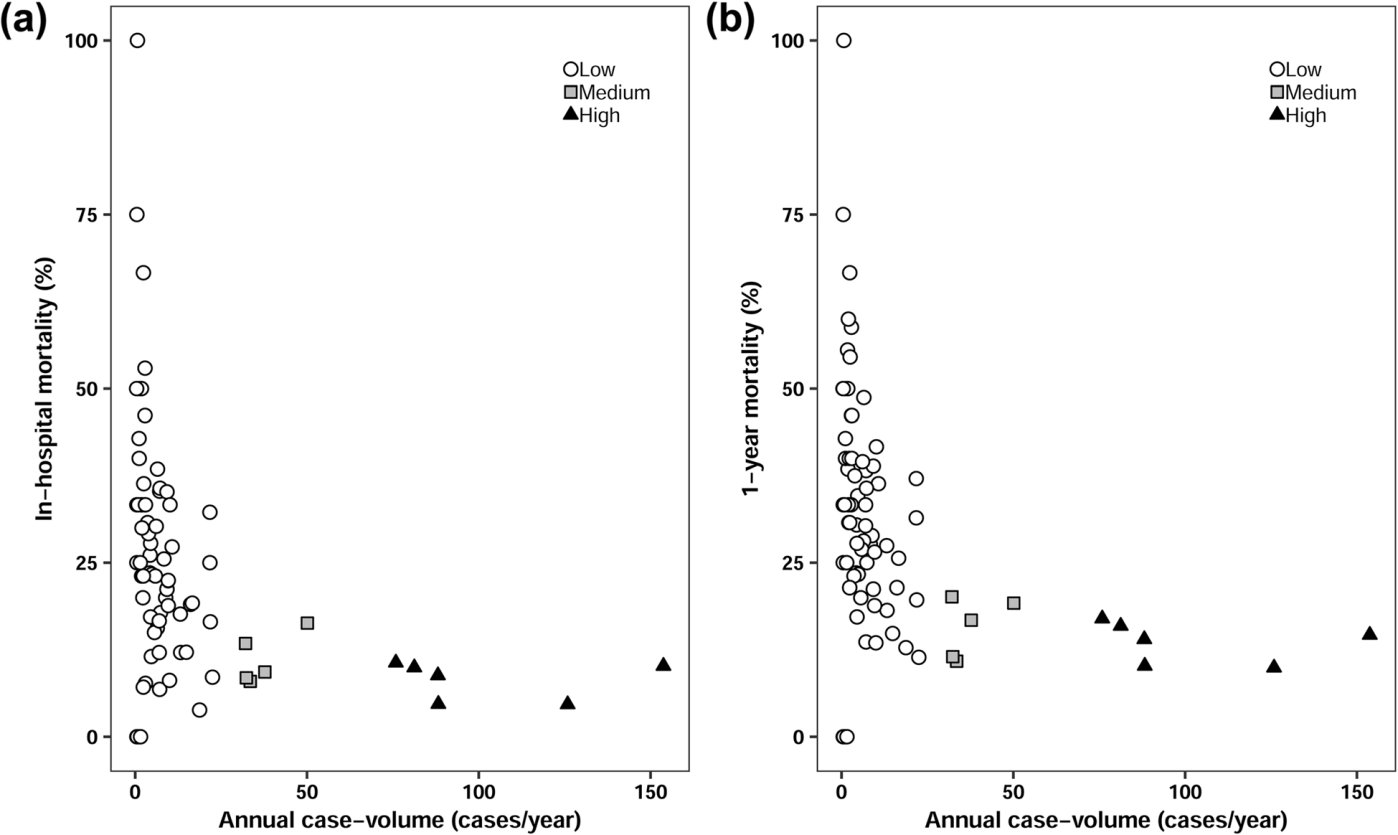
(**a**) In-hospital and (**b**) 1-year mortality after thoracic aorta replacement according to institutional case volume

The overall in-hospital mortality was 14.2% (690/4867). The in-hospital mortality in high, medium, and low volume centers were 8.6% (191/2222), 10.7% (77/717), and 21.9% (422/1928), respectively. Figure 1a shows the in-hospital mortality of each center based on their case volume. The risk of in-hospital mortality was significantly higher in the medium (the adjusted odds ratio [OR], 1.56; 95% confidence interval [CI], 1.16–2.11, *P* = 0.004) and low volume centers (the adjusted OR, 3.12; 95% CI, 2.54–3.85, *P* < 0.001) compared to high volume centers (Table 2).

**Table 2:** Logistic regression for in-hospital mortality after thoracic aorta replacement surgery

	Univariable model		Multivariable model	
	OR [95% CI]	*P*	OR [95% CI]	*P*
Case volume strata				
High volume (> 60 cases/year)	Reference		Reference	
Medium volume (30–60 cases/year)	1.28 [0.97–1.69]	0.084	1.56 [1.16–2.11]	0.004
Low volume (< 30 cases/year)	2.98 [2.48–3.58]	< 0.001	3.12 [2.54–3.85]	< 0.001
Age				
≤ 49	Reference		Reference	
50–59	1.11 [0.82–1.52]	0.502	1.14 [0.82–1.58]	0.449
60–69	1.51 [1.15–1.97]	0.003	1.51 [1.12–2.03]	0.008
70–79	2.17 [1.68–2.79]	< 0.001	2.21 [1.64–2.99]	< 0.001
≥ 80	2.69 [1.92–3.76]	< 0.001	3.30 [2.25–4.85]	< 0.001
Female	1.02 [0.87–1.20]	0.819	0.80 [0.66–0.97]	0.021
Extracardiac arteriopathy	1.30 [1.03–1.62]	0.024	1.08 [0.84–1.39]	0.559
Renal impairment	3.46 [2.09–5.75]	< 0.001	1.88 [1.07–3.33]	0.029
Chronic lung disease	1.21 [1.02–1.43]	0.028	0.96 [0.79–1.16]	0.649
Hypertension	1.19 [1.01–1.41]	0.038	0.89 [0.72–1.09]	0.262
Hyperlipidemia	1.14 [0.95–1.37]	0.164	1.03 [0.83–1.29]	0.776
Diabetes	1.33 [1.01–1.75]	0.042	1.01 [0.74–1.38]	0.933
Angina pectoris	1.25 [1.03–1.50]	0.023	1.12 [0.89–1.39]	0.342
Recent MI	1.51 [0.88–2.58]	0.135	1.19 [0.66–2.14]	0.560
History of PCI	2.45 [1.31–4.58]	0.005	1.64 [0.80–3.39]	0.181
Congestive heart failure	1.42 [1.09–1.85]	0.010	1.13 [0.83–1.53]	0.428
Emergent surgery	2.02 [1.45–2.82]	< 0.001	1.58 [1.09–2.31]	0.017
Atrial fibrillation	1.46 [1.04–2.06]	0.030	1.30 [0.89–1.90]	0.181
Perioperative RBC Transfusion				
0–1 units	Reference		Reference	
2–3 units	2.51 [1.27–4.95]	0.008	1.98 [0.99–3.96]	0.053
4–5 units	5.25 [2.65–10.38]	< 0.001	3.71 [1.86–7.43]	< 0.001
≥ 6 units	16.51 [8.39–32.47]	< 0.001	11.44 [5.75–22.79]	< 0.001
Surgery site				
Ascending aorta	Reference		Reference	
Aortic arch	1.89 [1.39–2.56]	< 0.001	1.55 [1.10–2.17]	0.012
Descending thoracic aorta	1.07 [0.86–1.33]	0.558	1.25 [0.96–1.63]	0.091
Combined	0.87 [0.71–1.06]	0.155	1.03 [0.82–1.29]	0.814
Surgery year	0.98 [0.94–1.01]	0.200	0.98 [0.94–1.02]	0.218

*CI* Confidence interval, *MI* Myocardial infarction, *OR* Odds ratio, *PCI* Percutaneous coronary intervention, *RBC* Red blood cell

### One year mortality

The overall 1 year mortality rate after thoracic aorta replacement surgery was 19.7% (960/ 4867). One year mortality was 14.0% (312/2222), 15.5% (111/717), and 27.9% (537/1928) in high, medium, and low volume centers, respectively. The distribution of 1 year mortality according to institutional case volume are shown in Fig. 1b. Compared to high volume center, the adjusted OR of 1-year mortality in medium volume center was 1.45 (95% CI, 1.12–1.87; *P* = 0.005; Table 3). In low volume center, the adjusted OR was 2.62 (95% CI, 2.18–3.14; *P* < 0.001; Table 3).

**Table 3:** Logistic regression for 1-year mortality after thoracic aorta replacement surgery

	Univariable model		Multivariable model	
	OR [95% CI]	*P*	OR [95% CI]	*P*
Case volume strata				
High volume (> 60 cases/year)	Reference		Reference	
Medium volume (30–60 cases/year)	1.12 [0.89–1.42]	0.340	1.45 [1.12–1.87]	0.005
Low volume (< 30 cases/year)	2.36 [2.02–2.76]	< 0.001	2.62 [2.18–3.14]	< 0.001
Age				
≤ 49	Reference		Reference	
50–59	1.16 [0.88–1.53]	0.278	1.23 [0.92–1.65]	0.168
60–69	1.63 [1.29–2.08]	< 0.001	1.72 [1.32–2.25]	< 0.001
70–79	2.53 [2.02–3.16]	< 0.001	2.81 [2.15–3.68]	< 0.001
≥ 80	3.62 [2.70–4.87]	< 0.001	5.05 [3.58–7.12]	< 0.001
Female	0.94 [0.82–1.08]	0.397	0.69 [0.58–0.82]	< 0.001
Extracardiac arteriopathy	1.42 [1.17–1.73]	< 0.001	1.18 [0.94–1.47]	0.157
Renal impairment	4.06 [2.50–6.58]	< 0.001	2.37 [1.37–4.08]	0.002
Chronic lung disease	1.28 [1.10–1.48]	< 0.001	1.00 [0.84–1.18]	0.980
Hypertension	1.27 [1.10–1.48]	0.001	0.94 [0.78–1.13]	0.520
Hyperlipidemia	1.18 [1.01–1.38]	0.048	1.02 [0.83–1.24]	0.882
Diabetes	1.39 [1.09–1.77]	0.008	1.01 [0.76–1.33]	0.957
Angina pectoris	1.20 [1.02–1.42]	0.030	1.00 [0.82–1.22]	0.994
Recent MI	1.50 [0.92–2.43]	0.101	1.14 [0.66–1.95]	0.643
History of PCI	2.61 [1.46–4.66]	0.001	1.76 [0.90–3.44]	0.101
Congestive heart failure	1.37 [1.08–1.74]	0.010	1.09 [0.83–1.44]	0.527
Emergent surgery	1.78 [1.31–2.43]	< 0.001	1.50 [1.05–2.13]	0.026
Atrial fibrillation	1.34 [0.98–1.83]	0.069	1.08 [0.76–1.54]	0.655
Perioperative RBC Transfusion				
0–1 units	Reference		Reference	
2–3 units	2.01 [1.21–3.34]	0.007	1.56 [0.93–2.63]	0.092
4–5 units	4.09 [2.46–6.80]	< 0.001	2.89 [1.71–4.87]	< 0.001
≥ 6 units	13.31 [8.03–22.08]	< 0.001	9.15 [5.43–15.42]	< 0.001
Surgery site				
Ascending aorta	Reference		Reference	
Aortic arch	1.98 [1.49–2.62]	< 0.001	1.63 [1.19–2.23]	0.002
Descending thoracic aorta	1.20 [0.98–1.46]	0.074	1.36 [1.07–1.72]	0.011
Combined	1.04 [0.87–1.24]	0.675	1.20 [0.98–1.47]	0.075
Surgery year	0.97 [0.94–1.00]	0.057	0.96 [0.93–0.99]	0.015

*CI* Confidence interval, *MI* Myocardial infarction, *OR* Odds ratio, *PCI* Percutaneous coronary intervention, *RBC* Red blood cell

### Cumulative all-cause mortality

**Fig. 2:**
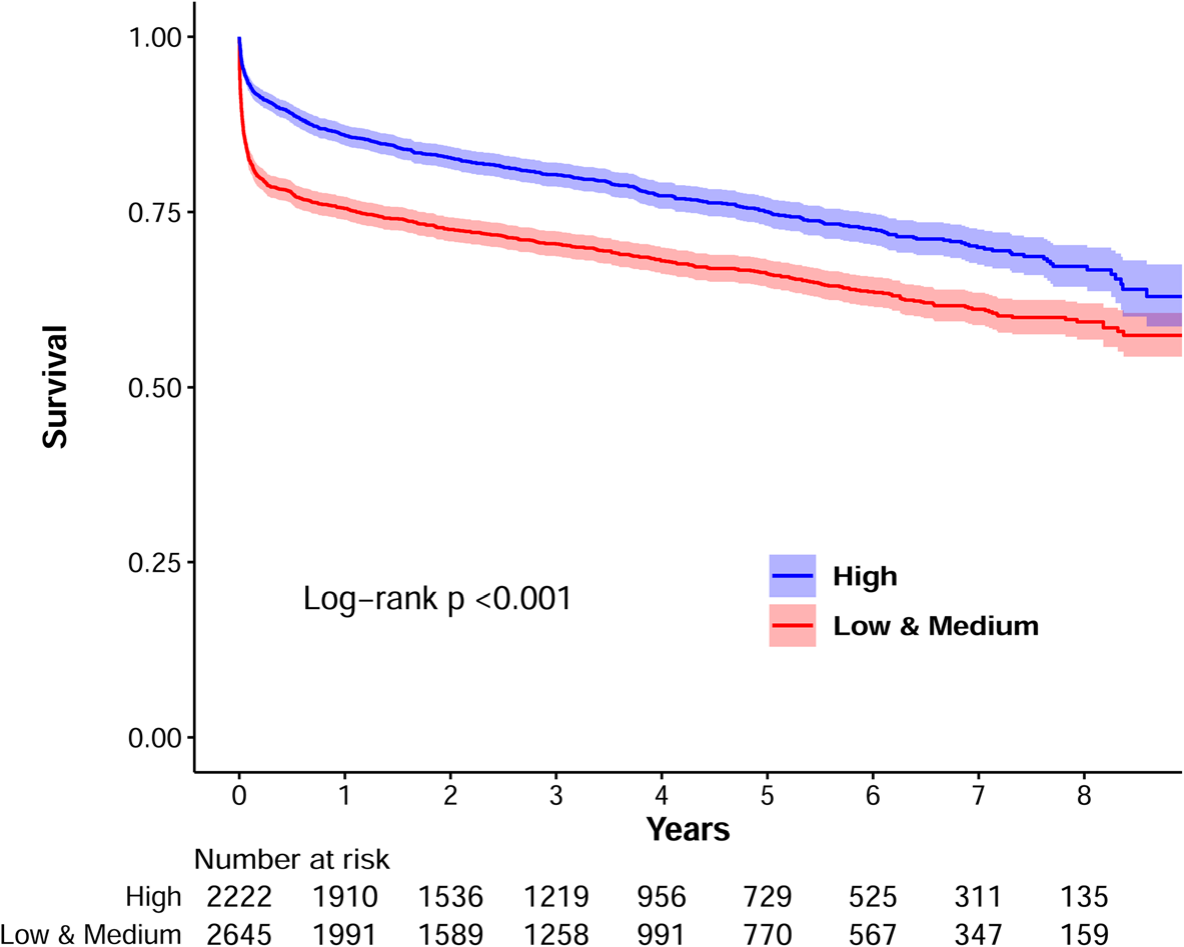
Kaplan-Meier curves of cumulative all-cause mortality after thoracic aorta replacement according to institutional case volume. The shaded area refers to 95% confidence interval

The results of Cox regression for cumulative all-cause mortality are presented in Table 4. The median (interquartile range) duration of follow-up after surgery was 3.1 (1.3–5.7) years. Low and medium volume centers were combined for the Cox regression analysis because the proportional hazards assumption was not met between the two groups. Patients who underwent thoracic aorta replacement surgery in low or medium volume centers showed a significantly higher risk of cumulative all-cause mortality compared to patients in high volume centers (adjusted hazard ratio, 1.55; 95% CI, 1.38–1.74; *P* < 0.001) (Table 4). The Kaplan-Meier survival curves with a follow-up period of up to 9 years showed a similar pattern (log-rank test, *P* < 0.001; Fig. 2).

**Table 4:** Cox proportional hazard model for cumulative all-cause mortality after thoracic aorta replacement surgery

	Univariable model		Multivariable model	
	HR (95% CI)	*P*	HR (95% CI)	*P*
Case volume strata				
High volume (> 60 cases/year)	Reference		Reference	
Low & medium volume (≤60 cases/year)^a^	1.53 (1.37–1.70)	< 0.001	1.55 (1.38–1.74)	< 0.001
Age				
≤ 49	Reference		Reference	
50–59	1.23 (0.98–1.53)	0.070	1.27 (1.02–1.58)	0.036
60–69	1.80 (1.49–2.18)	< 0.001	1.87 (1.54–2.28)	< 0.001
70–79	3.08 (2.58–3.67)	< 0.001	3.24 (2.67–3.93)	< 0.001
≥ 80	4.22 (3.39–5.25)	< 0.001	4.70 (3.73–5.93)	< 0.001
Female	0.97 (0.88–1.08)	0.621	0.73 (0.65–0.82)	< 0.001
Extracardiac arteriopathy	1.43 (1.24–1.65)	< 0.001	1.15 (1.00–1.33)	0.058
Renal impairment	3.02 (2.23–4.09)	< 0.001	1.89 (1.39–2.58)	< 0.001
Chronic lung disease	1.34 (1.20–1.49)	< 0.001	1.02 (0.91–1.14)	0.701
Hypertension	1.29 (1.16–1.44)	< 0.001	0.94 (0.83–1.06)	0.320
Hyperlipidemia	1.25 (1.11–1.41)	< 0.001	1.01 (0.89–1.15)	0.896
Diabetes	1.49 (1.25–1.76)	< 0.001	1.07 (0.90–1.28)	0.448
Angina pectoris	1.23 (1.09–1.39)	0.001	0.99 (0.87–1.13)	0.852
Recent MI	1.43 (1.02–2.01)	0.040	1.22 (0.86–1.73)	0.269
History of PCI	1.94 (1.28–2.93)	0.002	1.05 (0.68–1.61)	0.829
Congestive heart failure	1.44 (1.22–1.70)	< 0.001	1.17 (0.98–1.40)	0.081
Emergent surgery	1.35 (1.07–1.71)	0.012	1.37 (1.07–1.75)	0.012
Atrial fibrillation	1.45 (1.17–1.81)	0.001	1.17 (0.93–1.46)	0.178
Perioperative RBC Transfusion				
0–1 units	Reference		Reference	
2–3 units	2.32 (1.56–3.44)	< 0.001	1.80 (1.21–2.67)	0.004
4–5 units	3.76 (2.52–5.59)	< 0.001	2.74 (1.83–4.08)	< 0.001
6- units	9.02 (6.09–13.37)	< 0.001	5.95 (4.00–8.85)	< 0.001
Surgery site				
Ascending aorta	Reference		Reference	
Aortic arch	1.72 (1.41–2.09)	< 0.001	1.46 (1.20–1.79)	< 0.001
Descending thoracic aorta	1.10 (0.95–1.27)	0.193	1.25 (1.06–1.46)	0.006
Combined	1.02 (0.90–1.16)	0.760	1.08 (0.94–1.23)	0.287

*CI* Confidence interval, *HR* Hazard ratio, *MI* Myocardial infarction, *PCI* Percutaneous coronary intervention, *RBC* Red blood cell

^a^Low- and medium-volume groups were merged into one group prior to the multivariable analysis to meet the proportional hazard assumption

## Discussion

In this nationwide population-based study, low institutional case volume was an independent risk factor of mortality following thoracic aorta replacement surgery. The risk of in-hospital mortality was significantly higher in patients who underwent surgery in low (< 30 cases/year) and medium volume centers (30–60 cases/year), compared to high volume centers (> 60 cases/year). One year and cumulative all-cause mortality rates were both similarly higher in centers with low volume.

First described in the aircraft industry [14], the positive relationship between higher institutional case volume and improved patient survival have been consistently and repeatedly shown in high risk complex surgical procedures such as hepatectomy [4], esophagectomy [4, 5], lung resection [5, 15], and pelvic exenteration [4]. Thoracic aorta surgery is also a high-risk surgical procedure which requires complex and skilled surgical technique and immaculate perioperative care for best possible outcome. The reported incidence of operative mortality and major complications including stroke, infection, and renal failure following emergent surgical repair of acute thoracic or thoracoabdominal aortic dissection are exceptionally high, often exceeding 20 and 70%, respectively [16].

Numerous studies in cardiac surgery have shown that the risk of postoperative death was lower in high volume centers compared to lower volume centers including coronary artery bypass grafting [7], aortic valve replacement [17], mitral valve procedures [18], aortic root replacement [19], and heart transplantation [2]. A similar volume-outcome relationship have been reported in urgent or emergent abdominal aorta surgery [16, 20], but the relationship was between surgeon case volume, not institutional case volume, and patients outcome. The suggested cutoff was 10 ruptured abdominal aorta repairs and interestingly, there was no relationship between center volume and mortality [20]. Similarly, a previous national study in the United States revealed that the risk of mortality after emergent repair of thoracic or thoracoabdominal aortic dissection doubled in patients operated on by lower volume surgeons and centers (first quartile) compared to the highest volume surgeons [16]. The inverse association between institutional case volume and postoperative mortality was also noted in elective aortic root replacement surgery [19]. Our study included all types of thoracic aorta surgery and showed that the risk of postoperative death decreased significantly as institutional case volume increased.

Regionalization in the medical field is an attempt to concentrate resources to a few specialized health care centers /providers, often with an aim to improve patient outcome [21]. With a few exceptions such as in bariatric surgery [22], the literature in general tends to favor regionalization as shown in neonatal intensive care units [23] and designated pediatric trauma centers [24]. One recent relevant example may be the study which showed profound survival benefit in patients with influenza A-related (H1N1) acute respiratory distress syndrome after transfer to centers capable and experienced in extracorporeal membrane oxygenation [25]. A downside of regionalization may be decreased accessibility as shown in a simulated regionalization in pediatric cardiac surgery in the United States by closure of low volume hospitals which reduced postoperative mortality [26, 27]. Considering that previous studies were mostly performed in large countries, regionalization or concentration of high-risk cardiovascular surgeries to a limited number of select centers may be very effective for outcome optimization especially in relatively smaller countries where decreased geographical accessibility is negligible.

There are several limitations in our study that should be considered. First, although all cases of adult thoracic aorta replacement surgery performed during the past 8 years in Korea was included, bias may have been introduced due to the retrospective nature of the study design. Second, potential confounders such as laboratory data or clinical variables could not be obtained since the NHIS database was an administrative database in nature. Third, the information on the severity of thoracic aorta disease was lacking and may have affected postoperative patient outcome. Although a study suggested that the surgical indication for aorta surgery (dissection/ruptured aneurysm vs. intact aneurysms) had little effect on long-term mortality for 30-day survivors [28], another study suggested that in-hospital mortality seems to be worse in patients with ruptured thoracic aortic aneurysms compared to patients with intact thoracic aortic aneurysms [29]. Fourth, individual surgeon volume was not analyzed. Considering that most centers in Korea, including high volume centers, have a very limited number of surgeons who perform thoracic aorta surgery, the impact of institutional case volume on surgical outcomes may be comparable to that of surgeon volume.

## Conclusions

In conclusion, patients who underwent thoracic aorta replacement surgery in lower volume centers had significantly higher risk of in-hospital, 1-year, and cumulative all-cause mortality compared to patients in higher volume centers. However, considering the emergent nature of some thoracic aorta replacement surgeries and the different accessibility to institutions competent of performing the surgery, factors other than case volume should be considered when interpreting our results.

## Data Availability

The data that support the findings of this study are available from the National Health Insurance Service of Korea but restrictions apply to the availability of these Korean administrative data, which were used under license for the current study, and so are not publicly available.

## Abbreviations

CI: Confidence interval
ICD-10: International Classification of Diseases 10th revision
NHIS: National Health Insurance Service
OR: Odds ratio
